# Prophylactic central lymph node dissection in cN0 papillary thyroid cancer: a comparative study of via breast and transoral approach versus via breast approach alone

**DOI:** 10.3389/fendo.2024.1356739

**Published:** 2024-05-07

**Authors:** Rongliang Qiu, Jinbo Fu

**Affiliations:** ^1^ The School of Clinical Medicine, Fujian Medical University, Fuzhou, China; ^2^ Department of General Surgery, Zhongshan Hospital, Xiamen University, Xiamen, China

**Keywords:** papillary thyroid cancer, via breast and transoral approach, via breast approach alone, central lymph node dissection, thyroidectomy

## Abstract

**Background:**

Papillary thyroid cancer (PTC) progresses slowly and has a good prognosis, while the prognosis is worse if combined with central neck lymph node metastasis at an early stage. The different endoscope approaches may affect the thoroughness of lymph node dissection. This study aimed to compare the clinical efficacy and safety of prophylactic central lymph node dissection(CLND) for cN0 PTC performed via breast and transoral approach versus via breast approach alone.

**Materials and methods:**

A retrospective analysis of the surgical data of 136 patients with stage cN0 PTC was performed from August 2020 to December 2022. Among them, 64 underwent the breast and transoral approach (combined approach group), and 72 underwent the breast approach alone (breast approach group). The relevant indexes of surgery, the number of lymph nodes dissected, the occurrence of postoperative complications, and the cosmetic satisfaction of incision were statistically compared between the two groups.

**Results:**

The operation time of the combined approach group was 156.4 ± 29.8 min, significantly longer than that of the breast approach group, 119.6 ± 55.9 min, and the difference was statistically significant (P<0.05). The two groups of patients were compared in terms of intraoperative bleeding, postoperative drainage, hospitalization time, incision cosmetic satisfaction, and the occurrence of postoperative complications, and the differences were not statistically significant (P>0.05). The total number of lymph nodes retrieved in the central area (10.6 ± 7.1) and the number of positive lymph nodes (4.6 ± 4.9) in the combined approach group were significantly more than those in the breast approach group (7.4 ± 4.8, 1.6 ± 2.7), and the difference was statistically significant (P<0.05). The difference between the two groups in terms of the number of negative lymph nodes was not statistically significant (P>0.05).

**Conclusions:**

The study demonstrated that choosing the breast combined transoral approach for prophylactic CLND of cN0 PTC could more thoroughly clear the central area lymph nodes, especially the positive lymph nodes, which could help in the evaluation of the disease and the guidance of the treatment, while not increasing the postoperative complications. It provides a reference for clinicians to choose the appropriate surgical approach and also provides new ideas and methods for prophylactic CLND in patients with cN0 PTC.

## 1 Introduction

Cancer, a significant global public health event, is the second leading cause of death in the United States. According to global cancer statistics, thyroid cancer, which has the highest incidence of oncologic diseases, has an estimated 43,720 new cases in 2023 (1, 2). In recent years, with the increasing popularity of diagnostic imaging and ultrasound-guided fine-needle aspiration biopsy, the prevalence and detection of thyroid cancer have increased significantly, and surgical intervention remains the first-line treatment strategy for patients with thyroid malignancies (3). With the continuous improvement of endoscopic examination instruments, the concept of minimally invasive cosmetic surgery, and the emergence of various surgical techniques, endoscopic thyroidectomy can now fully meet the needs of young patients for scarless neck surgery (4). However, while laparoscopic thyroid surgery is widely carried out in centers at home and abroad due to its advantages, such as minor trauma, naturally shielded incisions, and sound cosmetic effects, there are also some problems and controversies. For example, differentiated thyroid cancer has a higher rate of lymph node metastasis, and lymph node metastasis is associated with higher local recurrence and distant metastasis, becoming a problem that troubles thyroid surgeons (5). The relevance of cervical lymph node dissection in patients with clinically or imaging-evident lymph nodes is well established (6–8). The American Thyroid Association guidelines recommend routine prophylactic central lymph node dissection (CLND) in patients with the stage of T3 or T4 tumors because of the increased risk of nodule metastasis in patients with lesions 4 cm or more in diameter (9). However, nowadays, there is controversy over whether prophylactic CLND should be performed for no lymph node metastasis (i.e., cN0) papillary thyroid cancer (PTC). The purpose of this article is to explore the advantages and disadvantages of via breast and transoral approach versus via breast approach alone for laparoscopic central zone lymph node dissection for thyroid cancer in terms of surgical-related indexes, complication rates, the cosmetic satisfaction of incision and the number of lymph nodes dissected, and to report our main results and conclusions, to provide a reference basis for the clinicians.

## 2 Materials and methods

### 2.1 General information

**Figure 1:**
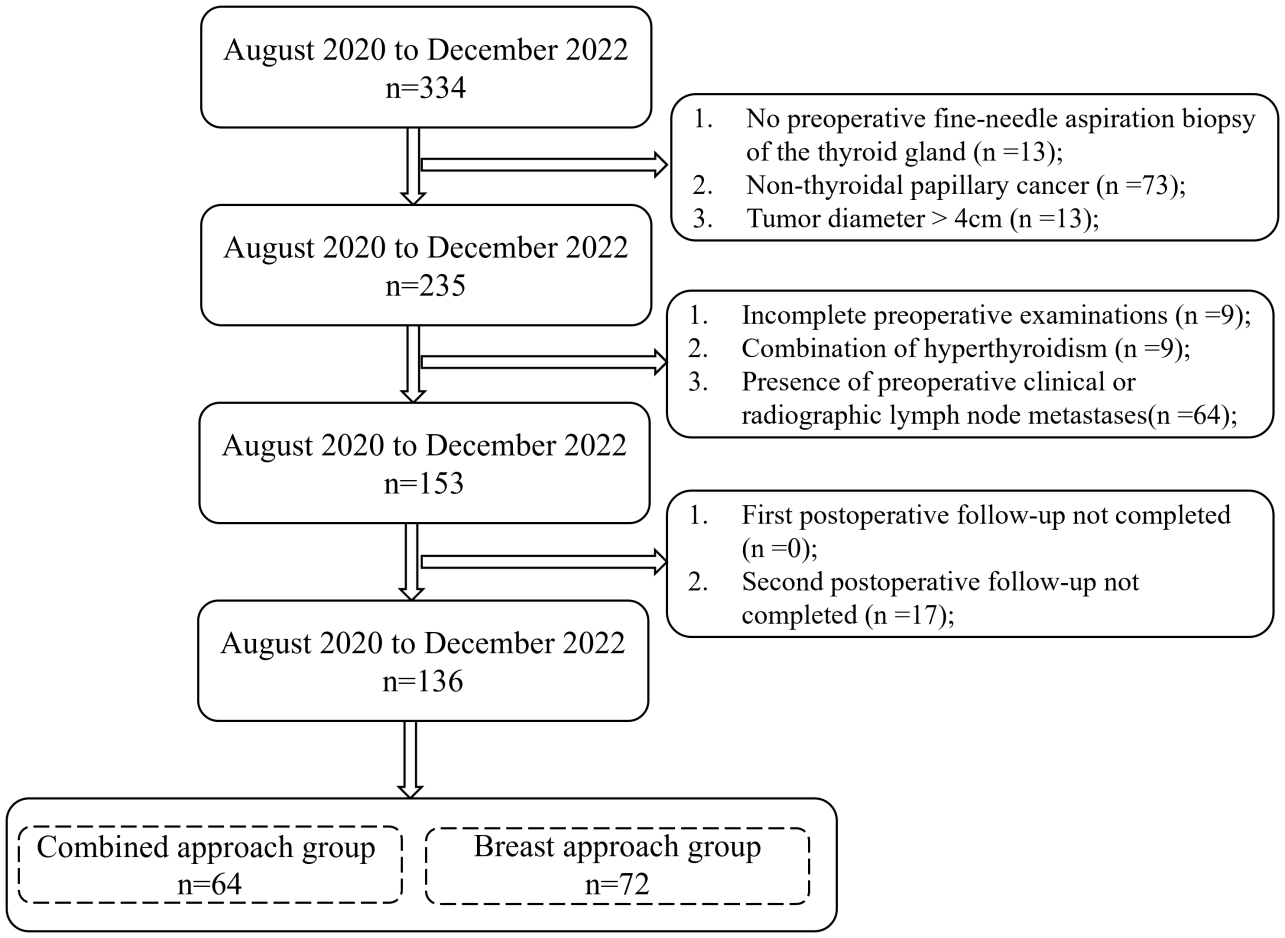
The flowchart of eligible patients.

334 thyroid patients admitted to the Department of General Surgery of Zhongshan Hospital of Xiamen University from August 2020 to December 2022 were retrospectively analyzed, and patients were screened according to the inclusion and exclusion criteria, finally, 136 patients with PTC were used as study subjects. The flowchart of eligible patients is shown in **Figure 1**. The surgical approach was selected according to the patients’ s wishes; 64 cases were in the combined approach group, which used the approach of breast combined with the transoral approach; 72 cases were in the breast approach group, which used the approach of breast approach alone. There were 18 males and 46 females in the combined approach group, aged 15-57 (35.3 ± 10.6), and 17 males and 55 females in the breast approach group, aged 20-65 (38.1 ± 8.6). Preoperative primary thyroid lesions were diagnosed by ultrasound-guided thyroid fine-needle aspiration pathology. There were preoperative neck or thyroid ultrasounds, cervical computed tomographic enhancement scans, laryngoscopy, and other related examinations. Postoperative pathology showed PTC. The general data of the two groups of patients, such as age, gender, body mass index (BMI), tumor size, tumor location, etc., were recorded. The difference between the general data of the two groups was not statistically significant (P>0.05) and was comparable. The details are shown in **Table 1**.

**Table 1:** Comparison of the general data between the two groups.

	Combined approach group (n=64)	Breast approach group (n=72)	Statisti*c*	*P* value
Mean Age (years)	35.3 ± 10.6	38.1 ± 8.6	-1.688	0.094
Age range (years)	15.0-57.0	20.0-65.0		
Sex			0.361	0.548
Female	46 (71.9)	55 (76.4)		
Male	18 (28.1)	17 (23.6)		
BMI (kg/m^2^)	23.2 ± 2.8	23.7 ± 3.5	-0.880	0.381
MLD (days)	30.0 (14.0-100.0)	35.5 (17.5-150.0)	-1.585	0.113
Tumor size (cm)	1.2 (0.8-2.2)	0.9 (0.7-1.6)	-1.654	0.098
Tumor location			4.373	0.112
Left	25 (39.1)	25 (34.7)		
Right	19 (29.7)	33 (45.8)		
Both	20 (31.3)	14 (19.4)		

BMI, body mass index; MLD, mean length of disease.

The Medical Ethics Committee of Zhongshan Hospital, Affiliated with Xiamen University, Xiamen, China, approved this study. As this study was retrospective, informed consent was not required.

### 2.2 Inclusion and exclusion criteria

The inclusion criteria were as follows: ①Preoperative ultrasound-guided fine-needle aspiration biopsy of the thyroid gland was performed to confirm the diagnosis of PTC; ②No obvious enlarged and fused lymph nodes in the central area on preoperative examination; no apparent enlarged lymph nodes in the upper mediastinum and lateral cervical area; ③Tumor diameter was less than 4 cm, and there was no invasion of the trachea, esophagus, and other vital surrounding tissues and structures.

The exclusion criteria were as follows: ① Those with a history of neck surgery or radiation; ② Those with coagulation and bleeding dysfunction; ③ Those with severe organic diseases who cannot tolerate surgery; ④ Those with hyperthyroidism or third-degree thyroid enlargement; ⑤ Pregnancy and lactation women.

### 2.3 Surgical procedures and postoperative management

A tracheal tube with nerve monitoring was placed via the oral route and fixed in the right corner of the patient’s mouth. General anesthesia was applied by tracheal intubation. The patient is placed in a natural supine position, with padding placed under the shoulders and a slight stretch of the neck. A prophylactic antibiotic course (Cefazolin Sodium, 2 g with 0.9% normal saline, 100 mL) was required to be started 30 min before surgery in the combined approach group and not in the breast approach group (10).

Thyroidectomy with prophylactic CLND by breast approach alone: The cervical thyroid surgery space was first established by the breast approach. A 1.0-cm incision was made at the midpoint before both nipples up to the depth of the deep fascial surface; the blunt stripping probe separated the deep and superficial fascia directly upward and outward until the lateral edge reached the line between the nipple and the outer edge of the sternocleidomastoid muscle, and the upper edge reached the lower edge of clavicle. After placement of a 10-mm trocar and endoscope, CO2 inflation was performed and the pressure was maintained at 8 mmHg to create a subcutaneous space. A 0.5-cm-long skin incision was made on each areola, a 5-mm trocar was inserted upward, and the Ultracision Harmonic scapel was directed upward to separate neck plasty deep surface and create an anterior cervical operating space in the deep layer of the platysma, which extended upward to the lower edge of the thyroid cartilage and laterally until the sternocleidomastoid muscle was exposed. After incising the white line of the neck through the breast approach, the chief surgeon separates the cervical muscles bilaterally and performs a thyroidectomy of the affected side or both sides with the Ultracision Harmonic scapel. The working space was then expanded to the lateral edge of the ipsilateral carotid sheath, up to the level of the hyoid bone. Lymph node dissection was performed in the central region (including the anterior larynx, anterior trachea, and paratrachea), and the recurrent laryngeal nerve was completely exposed. When the lower pole and paratracheal lymph nodes are removed, the lymph nodes and fat tissue should be pulled upward to the thymus. Finally, after careful inspection for surgical bleeding, the cervical white line was sutured and drains were placed.

Thyroidectomy with prophylactic CLND by combined breast and transoral approach: First, affected and bilateral thyroidectomies plus prophylactic CLND were performed as in the breast approach group. Then, the operating path of the transoral approach was established on the patient’s cephalic side. The three-hole approach method was used to make an incision of about 0.5 cm in length in each of the vestibule of the oral cavity and the lateral side of the cusp teeth on both sides, avoiding the chin nerve, and after blunt separation with scissors, three 5-mm Trocars were placed for downward puncture and placement into the anterior cervical operating space that had already been established by breast approach to continue the lymph node clearance in the remnant central area. Finally, the cervical white line was closed, and a drain was placed through the breast approach.

All patients were started on a liquid diet after 6 hours of postoperative fasting. A 3-day course of antibiotic prophylaxis (concentrated tinidazole gargles) was administered for the patients of the combined approach group, and none were needed in the breast approach group. Parathyroid hormone and serum calcium levels were routinely checked on the second postoperative day. The drain was removed when the postoperative drainage was less than 15 ml/d. Postoperative complications were graded according to the Clavien-Dindo classification (11) and included infection, postoperative bleeding, transient and permanent recurrent laryngeal nerve paralysis, transient and permanent hypoparathyroidism, accessory nerve injury, chyle leak, and so on. Complications were monitored for rapid recognition and prompt management. All patients received thyroid-stimulating hormone suppression therapy after surgery.

### 2.4 Observation indicators and fellow-up plan

Surgery-related indexes, including intraoperative blood loss, operation time, postoperative drainage, hospitalization time, and the number of lymph nodes dissected in the central area, were recorded in both groups to assess the clinical efficacy of the surgery. Follow-up observations were made and the occurrence of postoperative complications in both groups was recorded to assess the safety of the procedure.

The first follow-up of the patients was completed in the outpatient clinic at 1 month postoperatively. The second follow-up was at 6 months postoperatively, and patients were asked about their incision satisfaction and postoperative discomfort by questionnaire or telephone. The cosmetic satisfaction of the incision is evaluated using the visual analog scale. There are three levels of incision satisfaction: 0-3 points are dissatisfied, 4-6 points are generally satisfied, and 7-10 points are very satisfied.

### 2.5 Statistics

Data processing and analysis were completed using SPSS 26.0 statistical software. Measurement data were expressed as mean ± standard deviation(xˉ ± s). Normally distributed data were compared using the independent sample t-test, and non-normally distributed data were compared using the Humantney U test. Count data were expressed as n (%), and comparisons between groups were made using the chi-square or Fisher’s exact test. P<0.05 indicates that the difference is statistically significant.

## 3 Results

A total of 136 patients in both groups underwent successful completion of thyroidectomy and CLND without intermediate open thyroidectomy.

### 3.1 Surgical outcomes comparison

The operation time of the combined approach group was 156.4 ± 29.8 min, significantly longer than that of the breast approach group, 119.6 ± 55.9 min, and the difference was statistically significant (P<0.05). The difference was not statistically significant when comparing the two groups in terms of intraoperative blood loss, hospitalization time, and postoperative drainage (P>0.05). See **Table 2** for details.

**Table 2:** Surgical outcomes comparison of the two groups.

	Combined approach group (n=64)	Breast approach group (n=72)	*Z*	*P* value
Operation time (minutes)	156.4 ± 29.8	119.6 ± 55.9	4.854	<0.001
Intraoperative blood loss (ml)	31.3 ± 10.9	32.4 ± 9.2	-0.683	0.496
Postoperative drainage (ml)	152.3 ± 45.7	155.3 ± 80.1	-0.266	0.791
Hospitalization time (days)	7.3 ± 2.2	7.7 ± 2.5	-1.055	0.293

In the combined approach group, there were 52 cases of metastatic lymph nodes (52/64, 81.3%), and the total lymph nodes retrieved from the central area were 10.6 ± 7.1, of which 6.0 ± 5.1 were negative and 4.6 ± 4.9 were positive lymph nodes; Thirty cases in the breast approach group showed metastasis (30/70, 42.9%), and the total lymph nodes retrieved from the central area were 7.4 ± 4.8, of which 5.6 ± 4.5 were negative and 1.6 ± 2.7 were positive lymph nodes. The combined approach group cleared more total lymph nodes and more positive lymph nodes in the central area than the breast approach group, and the difference was statistically significant (P<0.05); the number of negative lymph nodes cleared was not statistically significant when comparing the two groups (P>0.05). For details, see **Table 3**.

**Table 3:** Pathological results of lymph node dissection of the two groups.

CLND	Combined approach group (n=64)	Breast approach group (n=72)	*t*	*P* value
Negative LN	6.0 ± 5.1	5.6 ± 4.5	0.510	0.611
Positive LN	4.6 ± 4.9	1.6 ± 2.7	4.350	<0.001
Total Retrieved LN	10.6 ± 7.1	7.4 ± 4.8	3.003	0.003

LN, lymph node; CLND, central lymph node dissection;.

### 3.2 Complications comparison

In assessing the safety of the procedure, the results related to postoperative complications and other related outcomes were compared between the two groups. There was no statistically significant difference between the combined approach group and the breast approach group in terms of postoperative complications such as infection, postoperative bleeding, transient and permanent recurrent laryngeal nerve palsy, transient and permanent hypoparathyroidism, accessory nerve injury, and chyle leak (P>0.05). For details, see **Table 4**.

**Table 4:** Comparison of the postoperative complications between the two groups.

	Combined approach group (n=64)	Breast approach group (n=72)	*χ²*	*P* value
Postoperative bleeding	0 (0.0)	0 (0.0)	–	–
TRLN	7 (10.9)	2 (2.8)	2.450	0.118
PRLN	3 (4.7)	0 (0.0)	1.620	0.203
TH	20 (31.3)	16 (22.2)	1.419	0.234
PH	0 (0.0)	0 (0.0)	–	–
Infection	1 (1.6)	1 (1.4)	–	>0.999
Accessory nerve injury	8 (12.5)	6 (8.3)	0.637	0.425
Chyle leak	3 (4.7)	3 (4.2)	<0.001	>0.999

TRLN, transient recurrent laryngeal nerve paralysis; PRLN, permanent recurrent laryngeal nerve paralysis; TH, transient hypoparathyroidism; PH, permanent hypoparathyroidism.

### 3.3 Follow-up results

**Figure 2:**
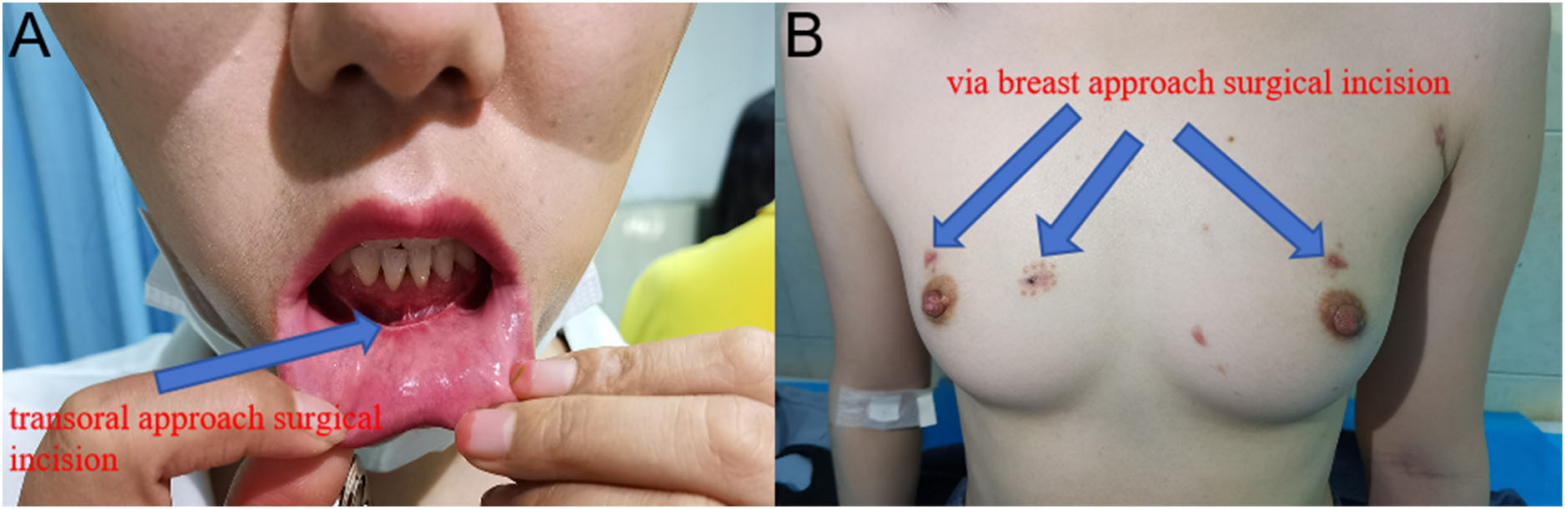
At one month postoperatively: pictures of the transoral approach and the breast approach incision. **(A)** Oral scar from the transoral approach. **(B)** Three incisions in the breast approach to thyroid surgery.

The cosmetic results were followed up at one month postoperatively, and a questionnaire scale was completed based on the patient’s satisfaction with the cosmetic results. It was found that the comparison of incision cosmetic satisfaction in the combined approach group compared to the breast approach group was not statistically significant (P>0.05). The incision score in the combined approach group (8.3 ± 1.1) compared to the breast approach group (7.9 ± 1.4), and the difference was not statistically significant (P>0.05). For details, see **Table 5** and **Figure 2**.

**Table 5:** Comparison of cosmetic effects of the two groups.

	Combined approach group (n=64)	Breast approach group (n=72)	*t* or *χ²*	*P* value
Incisional cosmetic satisfaction			3.745	0.053
Dissatisfaction	0 (0.0)	0 (0.0)		
General satisfaction	7 (10.9)	17 (23.6)		
Very satisfactory	57 (89.1)	55 (76.4)		
Cosmetic effect score	8.3 ± 1.1	7.9 ± 1.4	1.784	0.077

## 4 Discussion

As the most common endocrine tumor worldwide, the incidence of thyroid cancer is increasing year by year, among which PTC is the most common type, accounting for about 85-95% of overall thyroid cancers (12). Though PTC progresses slowly and has a good prognosis, it tends to have a poorer prognosis if combined with lymph node metastasis in the central neck at an early stage (13, 14). Currently, there is disagreement in the management of patients with clinically negative lymph nodes both at home and abroad, especially in the treatment of whether to perform prophylactic CLND for cN0 PTC. Different surgeons will adopt different management protocols. The 2015 version of the American Thyroid Association guidelines do not recommend routine prophylactic CLND, and its view is mainly based on the lack of sufficient evidence to support that prophylactic CLND is effective in reducing the recurrence rate and, on the contrary, may increase the potential surgical risk of patients (9, 12, 15, 16). However, Chinese guidelines for thyroid cancer have been advocating an aggressive treatment regimen, arguing that with preoperative and intraoperative risk assessment, as well as fine intraoperative dissection with effective preservation of the parathyroid glands and recurrent laryngeal nerves, prophylactic CLND along with resection of the primary lesion is justified because PTC lymph node metastasis is not uncommon (17). This study aimed to compare the difference between the combined approach group and the breast approach group during prophylactic CLND for cN0 PTC at the laparoscopic level.

Feasibility and safety are the primary considerations in evaluating surgical procedures. In our study, the two groups did not show statistical differences in terms of intraoperative blood loss, length of the hospital stay, postoperative drainage, and the occurrence of postoperative complications, and these results suggest that both approaches are safe and feasible. However, we noticed that the operation time was longer in the combined approach group compared to the breast approach group. The possible reasons for this are as follows: first, the combined approach group is based on the breast approach group with the addition of a transoral approach to complete the additional clearance of the central lymph nodes, which increased the operation time and difficulty to a certain extent. Second, the transoral approach involves dissecting the sub-chin region from the cephalad to the peduncle in a blind field, which requires the operator to have skillful surgical operation and rich anatomical knowledge, especially when dissecting the upper pole of the thyroid gland, preserving the parathyroid glands and the recurrent laryngeal nerve. Finally, the transoral approach has a small operating space, and the narrow operating space increases the possibility of collision between instruments, making the lens more susceptible to contamination, and frequent scrubbing of the lens increases the precision of the operation.

Lymph node clearance is another example of effectiveness. The number of CLND in the breast approach group in this study was 7.4 ± 4.8, which is comparable to the average CLND reported by other scholars [6.15 ± 4.90 (18), 7.3 ± 3.9 (19), and 7.53 ± 0.67 (20)], so it is reasonable to believe that the CLND by the breast approach achieves the same extent of dissection as that reported by scholars both nationally and internationally. However, we observed that the mean number of lymph nodes in the central area of the breast approach group was lower than that of traditional open surgery on the neck [10.87 ± 6.43 (21), 10.71 ± 5.17 (22), 10.1 ± 6.98 (23)]. This may be due to the presence of the sternal pedicle and clavicle obstructing the visual field and operating space in the breast approach group, which makes the CLND under complete endoscopic approach blind and may not be able to achieve complete clearance, especially in patients with preoperative upper mediastinal and retroclavicular lymph node enlargement, which is an absolute contraindication to this approach (24–26).

In this study, the mean number of CLND in the combined approach group was 10.6 ± 7.1, which was not different from that reported in previous studies [10.3 ± 4.6 (27), 11.3 ± 7.3 (28)]. At the same time, we observed no difference in the mean number of central lymph nodes cleared in the combined approach group compared with traditional open surgery on the neck [10.87 ± 6.43 (21), 10.71 ± 5.17 (22), 10.1 ± 6.98 (23)]. However, it is noteworthy that the combined approach group had a greater mean number of central zone lymph nodes compared to the transoral approach alone [7.00 ± 4.08 (29), 8.24 ± 4.88 (24), 6.1 ± 4.1 (30)]. In addition, our study also found that more central lymph nodes were cleared in the combined approach group than in the breast approach group, and the number of positive lymph nodes cleared was also higher. This finding may reveal the advantages of the transoral approach over other endoscopic approaches. The unique perspective of the transoral approach to endoscopic thyroid surgical operation can be taken into account from the cephalic side to the foot side, and there is no blindness to the retrosternal and retroclavicular operating space, which just makes up for the deficiencies that exist in the breast approach, as well as easier access to both the bilateral thyroid glands and the central compartments. In the combined approach group, the central lymph nodes were cleared through the transoral approach on top of the breast approach group, so more lymph nodes and positive lymph nodes were cleared in the central area, which was in line with our expectation, and it can be seen that the central lymph nodes of the breast approach are indeed challenging to be cleared entirely due to the existence of a blind zone. In addition, another advantage of the transoral approach is the complete scarlessness of the surface skin. Therefore, we believe that the combined approach can achieve good results in the radical treatment of tumors while taking into account the aesthetics.

The poor cosmetic outcome and long “L”-shaped or transverse curved neck scars resulting from traditional open neck surgery are important factors that contribute to the decreased quality of life of patients in the postoperative period (31). Therefore, while ensuring the safety and thoroughness of the surgery, choosing a less invasive thyroidectomy with a naturally obscured incision and a good cosmetic result can greatly improve the patient’s postoperative quality of life (32). There was no statistically significant difference in incision cosmetic satisfaction between the combined approach group compared to the breast approach group. Although the combined approach group has an increased surgical incision for the transoral approach compared to the breast approach group, the surgical incision for the transoral approach is completely obscured and completely scarless for thyroid surgery. However, surgical site infections are a special concern with transoral approach endoscopic thyroid surgery. Since the transoral approach involves the normal flora of the oral cavity, there is a risk of infection that will result in the conversion of a traditionally clean wound for thyroid surgery into a clean-contaminated wound (20, 33). Although previous studies have shown a low overall incidence of wound infections, prevention cannot be ignored (20). Therefore, in this study, prophylactic measures such as the use of specific antibiotics in the perioperative period and the administration of antibiotic mouth rinses for 3 days postoperatively were used to prevent postoperative infectious complications. Although no complications of postoperative infections occurred in this study, further studies are needed to determine the optimal dose and duration of antibiotics in the future. To minimize the occurrence of postoperative complications, based on adequate preoperative communication and evaluation, we performed fine dissection during the operation and used nerve monitoring instruments to monitor the recurrent laryngeal nerve, superior laryngeal nerve, vagus nerve, parasympathetic nerve, and phrenic nerve during the operation, to effectively preserve the parathyroid glands and nerves. In addition, postoperative complications are closely monitored for rapid recognition and timely management.

Although it is currently recognized that the central neck region is the first stop for cervical lymph node metastasis of thyroid cancer, i.e., the sentinel lymph nodes, followed by ipsilateral cervical zone lymph nodes, then contralateral cervical zone lymph nodes as well as mediastinal lymph nodes (15), however, international indications and technical specifications for endoscopic CLND for thyroid cancer are still lacking. Cervical CLND is the clearance of all lymph nodes adipose tissue in the paratracheal, anterior laryngeal, and paratracheoesophageal areas, i.e., posterior to the sternal stalk over the unnamed artery (34). Prophylactic sweeping during surgery must be performed thoroughly on the central zone lymph nodes according to the site of the tumor, which ranges from the lateral to the carotid sheath, the medial to the trachea, the superior border to the hyoid bone, and the inferior border to the unnamed artery (35). The difference between the anatomical structures of the right and left sides should be noted during the sweep to minimize the omission of lymph nodes that may metastasize.

The limitations of this study are the small sample size and short follow-up period, which failed to comprehensively assess the effects of the two surgical approaches on patients’ long-term prognosis and quality of survival. In the future, we will conduct a multicenter, large-sample, randomized, controlled clinical study to gain insight into the indications for prophylactic lymph node dissection in cN0 PTC.

## 5 Conclusion

This study is the first to compare the clinical efficacy and safety of prophylactic CLND for cN0 PTC performed via breast and transoral approach versus via breast approach alone and found that the combined approach group was able to more thoroughly clean the lymph nodes in the central area, especially the positive lymph nodes, which is beneficial for assessing the condition and guiding the treatment without increasing the incidence of postoperative complications. It provides a reference basis for clinicians to choose the appropriate surgical access and modality, as well as new ideas and methods for prophylactic CLND in patients with cN0 PTC.

## Data availability statement

The original contributions presented in the study are included in the article/Supplementary Material. Further inquiries can be directed to the corresponding author.

## Ethics statement

The studies involving humans were approved by The Medical Ethics Committee of Zhongshan Hospital, Affiliated with Xiamen University, Xiamen, China. The studies were conducted in accordance with the local legislation and institutional requirements. Written informed consent for participation in this study was provided by the participants’ legal guardians/next of kin. Written informed consent was obtained from the individual(s) for the publication of any potentially identifiable images or data included in this article.

## Author contributions

RQ: Conceptualization, Data curation, Methodology, Software, Writing – original draft, Writing – review & editing. JF: Conceptualization, Data curation, Writing – original draft, Writing – review & editing, Supervision, Validation.
